# Trends in treatment during the last stages of life in end-stage gynecologic cancer patients who received active palliative chemotherapy: a comparative analysis of 10-year data in a single institution

**DOI:** 10.1186/s12904-018-0348-7

**Published:** 2018-08-07

**Authors:** Tae-Kyu Jang, Dae-Yeon Kim, Shin-Wha Lee, Jeong-Yeol Park, Dae-Shik Suh, Jong-Hyeok Kim, Yong-Man Kim, Young-Tak Kim, Joo-Hyun Nam

**Affiliations:** Department of Obstetrics and Gynecology, Asan Medical center, University of Ulsan College of Medicine, 88 Olympic-ro 43-gil, Songpa-gu, Seoul 05505 South Korea

**Keywords:** Trend, The last stage of life, Gynecologic cancer, Active palliative chemotherapy

## Abstract

**Background:**

Palliative chemotherapy should be used with caution when attempting to alleviate symptoms in patients with end-stage cancer. However, palliative chemotherapy continues to be utilized in cancer patients during their last stages of life. In this study, we analyzed the pattern of chemotherapy administered during the last 6 months of life in patients with end-stage gynecologic cancer who were treated with active palliative chemotherapy for the past 10 years.

**Method:**

We retrospectively analyzed the data for patients with gynecologic cancer who died after undergoing active palliative chemotherapy without receiving hospice management at Asan Medical Center from 2006 to 2015. Patients were divided into two groups: those who died between 2006 and 2010, and those who died between 2011 and 2015. Based on the electronic medical records, the demographic and baseline characteristics of the patients, hospital admission during the last 6 months, invasive procedures, palliative chemotherapy patterns, and the time of the last chemotherapy session were confirmed.

**Results:**

A total of 193 patients with gynecologic cancer were eligible for this study. 92 patients died during 2006 to 2010, and 101 patients died during 2011 to 2015. The mean frequency of admission during the last 6 months was 5.12 for those who died in 2006–2010 and 6.06 for those who died during 2011–2015 (*p* = 0.003); similarly, the mean frequency of palliative chemotherapy during the last 6 months was 3.84 (2006–2010) vs. 4.93 times (2011–2015; *p* < 0.001). The proportion of patients undergoing invasive procedures during the last 3 months was 41.3% (2005–2010) vs. 56.4% (2011–2015; *p* = 0.044).

**Conclusions:**

The frequency of palliative chemotherapy and the rate of invasive procedures have increased in patients with end-stage gynecologic cancer who were treated aggressively without hospice management over 2011–2015 when compared to 2006–2010, along with an increase in the mean frequency of admission during the last 6 months at our institution. Gynecologic oncologists need to evaluate whether active palliative chemotherapy is beneficial to patients at the end-of-life stage, and if not helpful, should communicate with the patients and caregivers about when the palliative chemotherapy should be discontinued.

## Background

Palliative chemotherapy is indicated for patients with end-stage cancer for the purpose of alleviating life-threatening symptoms rather than cure, improving quality of life, and prolonging survival. This approach may be beneficial or harmful depending on the timing of use and/or the type of anticancer drug. However, oncologists tend to recommend continuous palliative chemotherapy for patients with end-stage cancer whose response rates are unclear [1]. One of the reasons for the increased use of palliative chemotherapy for end-stage cancer patients is the development of less toxic and better tolerated anticancer drugs. [1–3]. With the development of high efficacy, less-adverse anticancer drugs, clinicians including oncologists, have had many options for the use of anticancer drugs. In addition, oncologists are not always able to accurately predict the prognosis of all patients, but tend to be optimistic about the patient’s disease and prognosis. Lamont EB et al. reported that only 37% oncologists accurately predicted the actual prognosis; the rest overestimated [4]. Patients also tend to overestimate their life expectancy and misunderstand the purpose of palliative therapy, even after receiving detailed information from a clinician about their condition. This can make it easier for oncologists to choose an active palliative chemotherapy [5, 6].

Several studies have reported an excessive use of palliative chemotherapy for end-stage cancer patients. Liu et al. showed that the use of chemotherapy within the last month of life increased from 17.5% in 2001 to 21.0% in 2006. Other studies have reported that 9–43% of patients with end-stage cancer receive chemotherapy within the last 30 days of their lives [7–9]. Whether the use of palliative chemotherapy until the end of life would be beneficial for the patient remains unclear. This is because aggressive palliative chemotherapy may cause difficulties in identifying the appropriate time for hospice transfer and reduce the quality of life by increasing the re-hospitalization rate. Christakis et al. reported that hospice care should be initiated at least three months prior to receiving appropriate end-of-life management [10]. Keam et al. reported shorter survival durations and more frequent hospitalizations in patients receiving end-of-life chemotherapy [11].

The increased use of aggressive palliative chemotherapy seems to be similar in patients with end-stage gynecologic cancer. Although various treatment attempts have been made for patients with refractory gynecologic cancer, including immunotherapy and targeted therapies, these patients continue to receive palliative chemotherapy as they progress to the terminal stage of the disease [12, 13]. Therefore, we hypothesized that patients with end-stage gynecologic cancer who do not want hospice care would be managed with more aggressive palliative chemotherapy. In the current gynecologic oncology literature, there is limited data regarding when and how palliative chemotherapy is administered at the last stages of life. We analyzed the treatment patterns of end-stage gynecologic cancer patients who received active palliative chemotherapy over the last 10 years at our institution. In order to confirm trend in the use of active palliative chemotherapy, a comparative study was conducted by dividing into two groups (2006–2010 vs. 2011–2015) based on 2010, when new cancer insurance policy was implemented and new anti-cancer drugs were begun to introduced in Korea.

## Methods

### Study design and patients

This study was conducted at the Asan Medical Center, a multidisciplinary tertiary hospital serving in the Republic of Korea that does not have an inpatient hospice unit. We retrospectively analyzed data for patients who were treated for end-stage gynecologic cancer and died between January 1st, 2006 and December 31st, 2015 using electronic medical records that contained inpatient and outpatient clinic charts. End-stage was defined as a progressive state with no response to curative chemotherapy or a life expectancy of less than 6 months with distant metastasis. Palliative chemotherapy was defined as treatment administered not for the purpose of cure but for the purpose of improving symptoms or prolonging life. Patients were divided into two groups for comparison: those who died between January 1st, 2006 and December 31st, 2010, and those who died between January 1st, 2011 and December 31st, 2015. The exclusion criteria were as follows: 1) patients younger than 18 years, 2) patients who died only after conservative treatment without palliative chemotherapy, and 3) patients who died owing to complications of a curative operation. In this study, “active palliative chemotherapy” and “aggressive palliative chemotherapy” are used synonymously and mean active treatment until the end of life.

### Data collection

Following institutional review board approval, patient demographic and clinical characteristics including age, marital status, parity, primary cancer, International Federation of Gynecology and Obstetrics (FIGO, revised 2009 staging system) stage, and Eastern Cooperative Oncology Group performance status (ECOG PS) score at the time of the last admission were obtained from the hospital electronic medical records. In addition, data regarding the reason for the last admission, type of treatment and chemotherapy agent administered for the last 6 months, intensive care unit admission, and imaging examinations in the last month were obtained from the electronic medical records. All patient information obtained from hospital medical records was coded and anonymized. The patients were divided into two groups according to their year of death (2006–2010 vs. 2011–2015). The frequency of admission, chemotherapy during the last 6 months, invasive procedures during the last 3 months, and date of last chemotherapy session before death were compared between the two groups.

### Statistical analysis

Our medical data are expressed as number, mean ± standard deviation, or percentage. Mean values in the two groups were compared using the Student’s t-test or the Mann-Whitney U-test. Frequency distributions were compared using the chi-squared test or Fisher exact test. The Chi-squared test and Fisher exact test were conducted to assess potential differences between the two groups. *P*-values < 0.05 were regarded as indicating statistical significance. Data were analyzed using SPSS version 19.0 (SPSS, Chicago, IL, USA).

## Results

### Overall findings

Over the 10-year period investigated, 213 patients died during treatment for gynecologic cancer at the Asan Medical Center without transfer to a hospice care unit. Of these patients, three were less than aged below 18 years old, twelve who underwent only conservative treatment without active palliative chemotherapy, and five who died due to complications of a curative operation after primary diagnosis of gynecologic cancer were excluded. A total of 193 patients fulfilled the eligibility criteria and were included in this study. Of the 193 deaths, 92 occurred between January 2006 and December 2010 and 101 occurred between January 2011 and December 2015.

### Basic characteristics of the patients

The basic information for the 193 patients is listed in Table 1. The mean age of the patients was 54.33 years (range, 25–80 years). Among the gynecologic malignancies, ovarian cancer was the most common diagnosis, accounting for 110 patients (57%), followed by cervical (20.2%) and uterine (19.7%) cancers. Moreover, 139 patients (72%) had III-IV FIGO stage disease and 126 patients (65.3%) showed a ECOG PS score of 0–1 at the last admission.

**Table 1:** Basic characteristics of end-stage gynecologic cancer patients

Parameter	Total (*N* = 193)
Age (years), SD	54.33 ± 11.36
Marital status	
Married	163 (84.5)
Non-married (single)	15 (7.8)
Widowed or divorced	15 (7.8)
Parity	
0	27 (14.0)
1	33 (17.1)
≥ 2	133 (68.9)
Primary cancer	
Ovarian cancer	110 (57.0)
Cervical cancer	39 (20.2)
Uterine cancer	38 (19.7)
Vagina or vulvar cancer	6 (3.1)
FIGO stage	
I	26 (13.5)
II	28 (14.5)
III	94 (48.7)
IV	45 (23.3)
ECOG PS score at the last admission	
0	72 (37.3)
1	54 (28.0)
2	35 (18.1)
3	20 (10.4)
4	12 (6.2)

Data are presented as mean (range) or *n* (%) unless otherwise specified

FIGO: International Federation of Gynecology and Obstetrics, ECOG PS: Eastern Cooperative Oncology Group performance status

An analysis of the admissions and treatment during end-stage disease are listed in Table 2. Gastrointestinal problems were the most common cause of the last admission (26.9%), followed by cardiovascular or pulmonary problems (20.2%) and infection (16.2%). Type of treatment during the last 6 months was chemotherapy alone in 155 patients (80.3%), followed by chemotherapy with radiation in 26 patients (13.5%) and chemotherapy with surgery in 12 patients (6.2%). Among the 12 patients who underwent surgery, the most common indication was gastrointestinal problems (9/12, 75%); 2 patients underwent pelvic exenteration. In the last month, 23 patients (11.9%) were admitted to the intensive care unit and 137 patients (71.0%) underwent imaging examinations. Computed tomography was the most frequently performed imaging study, accounting for 101 patients (73.2%), followed by ≥2 imaging studies in 27 patients (19.7%).

**Table 2:** Admission and treatment of end-stage gynecologic cancer patients

Parameter	Total (*N* = 193)
Reason for last admission	
Palliative procedure or surgery	28 (14.5)
Gastrointestinal symptoms	52 (26.9)
Cardiovascular or pulmonary symptoms	39 (20.2)
Infectious state	31 (16.2)
Uncontrolled pain	11 (5.7)
Hematologic instability	12 (6.2)
Neurologic symptom	7 (3.6)
Others	13 (6.7)
ICU admission in the last month	23 (11.9)
Imaging examination conducted in the last month	
No examination	56 (29.0)
Examination	137 (71.0)
CT	101 (73.7)
MRI	8 (5.8)
PET-CT	1 (0.7)
Combined imaging (> 2)	27 (19.7)
Type of treatment administered during the last 6 mo	
Chemotherapy alone	155 (80.3)
Chemotherapy with radiation	26 (13.5)
Chemotherapy with surgery	12 (6.2)
Gastrointestinal surgery	9
Pelvic exenteration	2
Neuro-surgery	1
Type of chemotherapy-agent administered during the last 6 mo	
IV only	177 (91.7)
IV with PO	16 (8.3)

Data are presented as *n* (%) unless otherwise specified

*ICU* intensive care unit, *CT* computed tomography, *MRI* magnetic resonance imaging, *PET-CT* positron emission tomography-computed tomography, *IV* intravenous, *PO* per os

### Comparison between the two groups (2006–2010 versus 2011–2015)

As shown in Table 3, there were no significant differences between patients who died during 2006–2010 and those who died during 2011–2015 in terms of basic characteristics. The total number of cases of palliative chemotherapy was not significantly different between the groups. The duration from the last admission to death (24.3 days vs. 27.0 days, *p* = 0.535) and the duration from the last chemotherapy session to death (47.0 days vs. 58.1 days, *p* = 0.067) were shorter among patients who died during 2011–2015 than in those who died during 2006–2010, but without statistical significance.

**Table 3:** Comparison between patients who died during 2006–2010 and those who died during 2011–2015

Parameter	2006–2010 (*N* = 92)	2011–2015 (*N* = 101)	*p*-value
Age (years), SD	53.2 ± 13.07	55.4 ± 9.48	0.192
Marital status			0.136
Married	74 (80.4)	89 (88.1)	
Non-married (single)	11 (14.0)	4 (4.0)	
Widowed or divorced	7 (7.6)	8 (7.9)	
Parity			0.180
0	17 (18.5)	10 (9.9)	
1	13 (14.1)	20 (19.8)	
≥ 2	62 (67.4)	71 (70.3)	
Primary cancer			0.350
Ovarian cancer	57 (62.0)	53 (52.5)	
Cervical cancer	16 (17.4)	23 (22.8)	
Uterine cancer	15 (16.3)	23 (22.8)	
Vagina or vulvar cancer	4 (4.3)	2 (2.0)	
FIGO stage			0.447
I	10 (10.9)	16 (15.8)	
II	16 (17.4)	2 (14.5)	
III	47 (51.1)	47 (46.5)	
IV	19 (20.7)	26 (25.7)	
ECOG PS score at the last admission			0.248
0	41 (44.6)	31 (30.7)	
1	24 (26.1)	30 (29.7)	
2	12 (13.0)	23 (22.8)	
3	10 (10.9)	10 (9.9)	
4	5 (5.4)	7 (6.9)	
Reason for last admission			0.775
Procedure or palliative treatment	16 (17.4)	12 (11.9)	
Gastrointestinal symptoms	25 (27.2)	27 (26.7)	
Cardiovascular or pulmonary symptoms	17 (18.5)	22 (21.8)	
Infectious state	11 (12.0)	20 (19.8)	
Uncontrolled pain	6 (6.5)	5 (5.0)	
Hematologic instability	7 (7.6)	5 (5.0)	
Neurologic symptoms	3 (3.3)	4 (4.0)	
Others	7 (7.6)	6 (5.9)	
ICU admission in the last month	11 (12.0)	12 (11.9)	0.987
Imaging examination conducted in the last month			0.011
No examination	35 (38.0)	21 (20.8)	
Examination (CT, MRI, PET-CT)	57 (62.0)	80 (79.2)	
Type of treatment administered during the last 6 mo			0.004
Chemotherapy alone	82 (89.1)	73 (72.3)	
Chemotherapy with radiation or surgery	10 (10.9)	28 (27.7)	
Type of chemotherapy-agent administered during the last 6 mo			0.199
IV only	87 (94.6)	90 (89.1)	
IV with PO	5 (5.4)	11 (10.9)	
Number of total palliative chemotherapy sessions	3.08	3.24	0.587
Frequency of admission during the last 6 mo	5.12	6.06	0.003
Frequency of chemotherapy during the last 6 mo	3.84	4.93	< 0.001
Invasive procedure during the last 3 mo	38 (41.3)	57 (56.4)	0.044
Duration from last admission to death (d)	27.0	24.3	0.535
Duration from last chemotherapy to death (d)	58.1	47.0	0.067
Date of last chemotherapy before death			0.486
Between the last 3 mo and 6 mo	20 (21.7)	18 (17.8)	
Between the last 1 mo and 3 mo	47 (51.1)	45 (44.6)	
Between the last 1 mo and 2 wk	12 (13.0)	19 (18.8)	
Within the last 2wk	12 (13.0)	20 (19.8)	

Data are presented as mean (range) or *n* (%) unless otherwise specified

Invasive procedure: paracentesis, thoracentesis, insertion or removal of catheters or stents in vessels or organs excluding the surgery

However, among patients who died during 2011–2015, the frequency of imaging examination in the last month was higher than that in patients who died during 2006–2010 (79.2% vs. 62.0%, *p* = 0.011). The frequency of chemotherapy with radiation or surgery during the last 6 months was higher in patients who died during 2011–2015 than in those who died during 2006–2010 (27.7% vs. 10.9%, *p* = 0.004). The mean frequency of admission (6.06 vs. 5.12, *p* = 0.003) and palliative chemotherapy (4.93 vs. 3.84, *p* < 0.001) during the last 6 months was higher among patients who died during 2011–2015 than among those who died during 2006–2010. Invasive procedures during the last 3 months were performed more frequently among patients who died during 2011–2015 than among those who died during 2006–2010 (56.4% vs. 41.3%, *p* = 0.044).

**Fig. 1:**
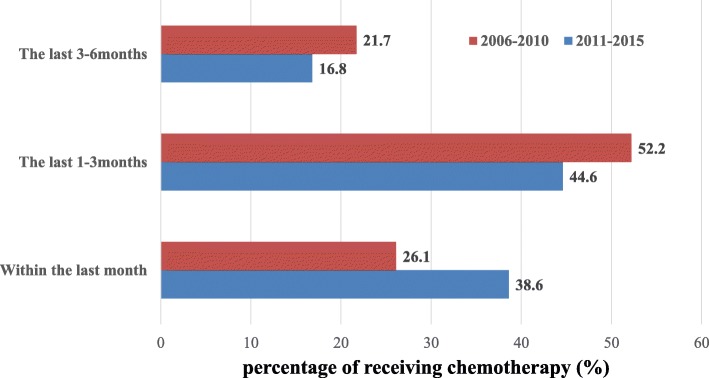
Comparison of time to receive chemotherapy during the last 6 months (2006–2010 vs. 2011–2015). Shows the percentage of patients treated with palliative chemotherapy during the last 6 months. From 2011 to 2015, it can be identified that more active palliative chemotherapy is performed until the end of life. It is notable that the percentage of patients receiving chemotherapy during the last month of life from 2011 to 2015 is not statistically significant, but is higher than 2006–2010 (38.6% vs. 26.1%, *p* = 0.068)

The date when chemotherapy was administered before death did not significantly differ between the groups; however, the rate of at least one palliative chemotherapy session administered within the last 30 days of life increased from 26.1% during 2006–2010 to 38.6% during 2011–2015 (*p* = 0.068; Fig. 1).

## Discussion

Quality of life is one of the most important components of the well-being of end-stage cancer patients. The use of chemotherapy in patients with end-stage cancer should involve a very cautious approach. During the last months of life, palliative chemotherapy can have a direct impact on quality of life because the toxic side effects of anticancer drugs can lead to a life-threatening situation; moreover, continuous treatment can deprive the patients of the opportunity to receive appropriate hospice care. In our study, 32.6% of the patients with end-stage gynecologic cancer were treated with active palliative chemotherapy within the last month of life and 16.6% during the last 2 weeks. Our results are not very different from those of other studies, with the frequency of active palliative chemotherapy administered within the last month ranging from 18 to 55.6%, and within the last 2 weeks from 5.3 to 33.8% [11, 14–17]. However, when we divided the data into 5-year periods, the rate of active palliative chemotherapy conducted within the last month increased from 26.1 to 38.6% over the last 5 years. As a result, the frequency of imaging examination in the last month of life increased from 62.0 to 79.2%, and the frequency of invasive procedures during the last 3 months increased from 41.3 to 56.4% over the last 5 years. These results suggest that patients with end-stage gynecologic cancer in the last 5 years have been aggressively treated with palliative chemotherapy until death. The application of aggressive palliative chemotherapy can also be confirmed by the frequency of admission and chemotherapy during the last 6 months. The frequency of admission and chemotherapy during the last 6 months increased from 5.12 to 6.06 and from 3.84 to 4.93, respectively. Apart from the application of aggressive palliative chemotherapy, a noteworthy result of our study is that the frequency of chemotherapy along with radiation or surgery administered to patients with end-stage gynecologic cancer, rather than chemotherapy alone, was higher in 2011–2015 than in 2006–2010 (13.5% vs. 27.7%). In other words, end-stage gynecologic cancer patients are being actively managed in more ways than ever before.

When we analyze the reasons for the recent increase in the use of aggressive palliative chemotherapy based on the results from our institution, the first is the development of new anticancer drugs and existing anticancer drugs over the past decade. The development of anticancer therapies has brought about higher efficacy and fewer side effects, which increases the use of palliative chemotherapy towards the end of life. For example, pegylated liposomal doxorubicin is thus an upgraded anticancer drug with a modified pharmacokinetic and safety profile when compared to conventional doxorubicin, and has been approved for use in Korea in both platinum-sensitive and resistant, recurrent ovarian cancer patients since 2014 [18–20]. Bevacizumab, a novel anticancer agent used widely in cases of gynecologic cancer, is a monoclonal antibody that targets vascular endothelial growth factor and has proven efficacy in platinum-resistant ovarian cancer, when administered as a single agent and in combination with cytotoxic chemotherapy [21–24]. In addition, combination therapy with bevacizumab was approved as a treatment for persistent, recurrent, or metastatic cervical cancer on the basis of the findings of an international phase 2 randomized trial [23]. In 2015, the expansion of insurance coverage to include cervical cancer and ovarian cancer in Korea has broadened the range of anticancer drug choices. Poly (ADP-ribose) polymerase (PARP) inhibitors associated with the repair of double-strand DNA breaks are currently undergoing various clinical trials as treatments for solid and hematological cancers. One such PARP inhibitors, olaparib, was approved as a monotherapy agent by the Food and Drug Administration in 2014 for the treatment of platinum-sensitive recurrent ovarian cancer with a germline BRCA 1/2 mutation; another type of PARP inhibitor, rucaparib, received Food and Drug Administration approval in 2016 [25, 26]. These newly developed or improved anticancer drugs can be used alone or in combination with existing anticancer drugs, which has broadened the selection of drugs available to gynecologic oncologists; the development of high efficacy and less toxic anticancer drugs is thought to be closely related to the recent application of an increasingly palliative anticancer drug.

Secondly, the revisions to the Korean insurance system in 2010 could also be a cause of the increased use of palliative chemotherapy. The main outcome of the revised insurance scheme is the reduced burden on the total amount of medical care allocated to cancer patients. The previous insurance system covered only 80% of the total medical amount for cancer patients. However, the revised insurance system covered up to 95% of the total medical amount, reducing the financial burden of cancer patients by 15%. As the burden of anticancer drug expenditure has decreased, the application of anticancer therapy has become easier for physicians now compared to that before 2009 in terms of economy.

How should we look at the use of increasingly active palliative chemotherapy in gynecologic cancer patients? The various palliative modalities themselves, including active palliative chemotherapy, surgery, and radiation therapy, is worth attempting in end-stage cancer patients. These modalities can improve the quality of life by improving the symptoms of end-stage cancer patients who seek active palliative treatment and have a positive attitude toward treatment. Radiation therapy is useful for palliative treatment for acute pain or bleeding due to metastatic lesions. And as mentioned above, new anticancer drugs have proven to have a significant survival benefit in gynecologic cancer patients. It is expected that the use of various palliative modalities will continue to increase as an important aspect of palliative treatment.

However, it is clear that palliative therapy is aimed at improving symptoms rather than prolonging the life span. For this reason, a cautious approach is needed when applying palliative care to the patient. Excessive palliative chemotherapy may have toxic effects that results in less time for appropriate hospice management for end-stage cancer patients. Although there is no definitive answer for when anticancer drugs should be administered in patients with end-stage cancer, it is necessary to reconsider the fact that the proportion of active palliative chemotherapy sessions in the last month of life is increasing like our results of study. Several studies have reported negative outcomes of aggressive treatment administered at the end of life, particularly with regard to quality of life. Wright reported that end-stage cancer patients who received active palliative treatment had poor quality of life and that their caregiver suffered from greater pain after the patient’s death [27]. Although the range of treatment is limited to palliative chemotherapy, negative opinions have become mainstream. In a study involving more than 600 cancer patients published by Lee in 2015, the overall survival rate was higher in patients receiving early palliative care services without active palliative chemotherapy [28]. Another study also reported that patients who received palliative chemotherapy during the last month of life had a significantly shorter survival duration (from palliative treatment to death) and more frequent hospitalizations [29].

Another issue to consider in the trend toward aggressive palliative chemotherapy is the economic burden. Even if the cost burden on the patient is reduced from 20 to 5% owing to the revised insurance system, a burden still exists. Frequent hospitalization inevitably leads to an increase in overall treatment costs such as counseling, medication, and imaging examination. Cheung et al. compared the mean overall hospitalization costs of a group of end-stage cancer patients who received aggressive care and a group of end-stage cancer patients who received non-aggressive care. Those who received aggressive care paid a mean $5453 more than the patients who received non-aggressive care; the hospitalization cost accounted for the largest difference [30]. Chastek et al. analyzed the cost of terminal cancer for 6 months before death and reported that the length of hospital stay accounted for the largest portion of hospitalization costs [31].

The recent increasing application of aggressive palliative chemotherapy and invasive procedures in end-stage gynecologic cancer patients needs to be reviewed by physicians managing cancer. Deciding on palliative chemotherapy for the end-of-life stage should be taken seriously in situations where the use of palliative chemotherapy is not clear enough to improve the patient’s quality of life or symptoms. Given the physical, psychological, and economic benefits of hospice care at the appropriate time, the potential benefits and drawbacks of palliative chemotherapy should be explained to the patient and caregivers in detail, and decisions should be mutually agreed upon. For example, our institution is operating a referral center in conjunction with a local hospice center to provide proper palliative care and conducting multidisciplinary care involving patients and caregivers for integrated management such as psychological, economic support, and discussion of treatment plan for end-stage cancer patients.

The strength of our study was the analysis of the patterns and trends of treatment for patients with end-stage gynecologic cancer who were treated with active palliative chemotherapy, excluding those who had undergone hospice care or conservative treatment. To our knowledge, there has been no comparative analysis of long-term (such as 10 years) treatment patterns of aggressive palliative chemotherapy in patients with gynecologic cancer. Conversely, there are some limitations in this study. As a retrospective study performed in a single institution, it is difficult to generalize the results of our study. Since this retrospective study targeted only patients who died after undergoing active palliative chemotherapy without hospice care in a tertiary hospital specialized in cancer treatment, it is difficult to conclude that the use of total palliative chemotherapy has increased in patients with end-stage gynecologic cancer due to selection bias. In addition, it has been confirmed that more aggressive palliative chemotherapy has been performed over the recent 5 years (2011–2015), but no further studies such as a comparison of the quality of life or overall survival of these patients have been conducted. Nevertheless, the results of this study can be used as data to confirm the trend of active palliative chemotherapy in end-stage gynecologic cancer patients; furthermore, the tendency toward palliative chemotherapy for all end-stage cancer patients is evident.

## Conclusion

The frequency of palliative chemotherapy and invasive procedures conducted at the end-stage gynecologic cancer patients who were treated aggressively without hospice management at our institution has increased over the recent 5 years compared to that in the past, along with an increase in the mean frequency of admission during the last 6 months. On the other hand, the duration from the last chemotherapy session to death has decreased. Gynecologic oncologists consistently need to assess whether active palliative chemotherapy is beneficial for patients with end-stage cancer. If active palliative chemotherapy has an uncertain benefit to the patient, it may be important to communicate with patients and caregivers about continuing use of anticancer drugs. To support our results, a large-scale multi-center comparative study will be required.
